# Effect of Magnesium Supplementation on Inflammatory Parameters: A Meta-Analysis of Randomized Controlled Trials

**DOI:** 10.3390/nu14030679

**Published:** 2022-02-05

**Authors:** Nicola Veronese, Damiano Pizzol, Lee Smith, Ligia J. Dominguez, Mario Barbagallo

**Affiliations:** 1 Geriatric Unit, Department of Internal Medicine and Geriatrics, University of Palermo, 90127 Palermo, Italy; nicola.veronese@unipa.it (N.V.); mario.barbagallo@unipa.it (M.B.); 2 Italian Agency for Development Cooperation, Khartoum 11115, Sudan; damianopizzol8@gmail.com; 3 Centre for Health, Performance and Wellbeing, Anglia Ruskin University, Cambridge CB1 1PT, UK; Lee.Smith@anglia.ac.uk; 4 Faculty of Medicine and Surgery, Kore University of Enna, 94100 Enna, Italy

**Keywords:** magnesium, inflammation, C reactive protein, tumor necrosis factor, randomized controlled trial, meta-analysis

## Abstract

Magnesium (Mg) may have several beneficial effects on human health outcomes. One hypothesized mechanism eliciting such effects is the action of Mg on serum inflammatory parameters. However, studies on this topic to date have several important limitations. Therefore, the present systematic review and meta-analysis aimed to summarize the current state of the art of all randomized control trials (RCTs) investigating the effects of Mg supplementation versus placebo on serum parameters of inflammation. We searched several databases until 23 November 2021 for RCTs. Eligible studies were RCTs investigating the effect of oral Mg supplementation vs. placebo and having serum inflammatory markers as an outcome. Among 2484 papers initially screened, 17 randomized controlled trials (889 participants; mean age: 46 years; females: 62.5%) were included. Generally, a low risk of bias was present. In meta-analysis, Mg supplementation significantly decreased serum C reactive protein (CRP) and increased nitric oxide (NO) levels. In descriptive findings, Mg supplementation significantly reduced plasma fibrinogen, tartrate-resistant acid phosphatase type 5, tumor necrosis factor-ligand superfamily member 13B, ST2 protein, and IL-1. In conclusion, Mg supplementation may significantly reduce different human inflammatory markers, in particular serum CRP and NO levels.

## 1. Introduction

The literature regarding the health benefits of magnesium (Mg) is exponentially increasing [1]. In an umbrella review with 16 meta-analyses and 50 independent outcomes findings suggested that Mg is associated with several positive health outcomes [1]. It is widely known that Mg is involved in more than 600 enzymatic reactions [2], consequently having a wide spectrum of actions in pregnancy [3,4,5], as well as in cardiovascular [6,7], gastrointestinal [8], infectious [9], and metabolic diseases [10], such as diabetes [11,12].

It should be acknowledged that several papers have reported that Mg has positive effects on medical events likely owing to improving inflammatory parameters [13,14,15]. For example, one observational study reported an inverse relationship between dietary magnesium intake and inflammatory parameter levels (in particular C-reactive protein, CRP) in people affected by obesity [13]. However, observational studies on this topic to date have been limited by research design or heterogeneity of the participants included (e.g., gender, ethnicity, age, etc.) and likely underpowered to achieve comprehensive and reliable conclusions [14,15]. Two meta-analyses have also shown that Mg supplementation can have differing effects on some indices of inflammatory and anti-inflammatory indexes, such as CRP [14,15]. Despite the importance of these two meta-analyses [14,15], many other studies are now available although the evidence is limited to CRP as an outcome.

Although the pathophysiological mechanisms through which Mg may improve inflammatory status is not clear yet, it has been demonstrated in, animal models and human studies that Mg deficiency acts as a trigger for the inflammatory process [15]. A possible explanation is that the reduction of Mg levels stimulates macrophages and influx of calcium ions into cells [16]. The increased cell calcium levels increase the Mg necessary to block the influx of calcium ions with an increased stimulation of N-methyl-D-aspartate receptors that present high permeability to calcium [16]. Thus, the stimulation of these receptors lead to the opening of non-selective channels to cations with a consequent rise of calcium ions in neuronal cells [16]. The result is the releasing of neurotransmitters and cytokines as IL-6 that, in turn, enhance CRP release starting the inflammatory response [17].

Given this background, the present systematic review and meta-analysis aimed to summarize the current state of the art of all randomized control trials (RCTs) investigating the effects of Mg supplementation versus placebo on serum parameters of chronic inflammation.

## 2. Materials and Methods

This systematic review adhered to the PRISMA statement [18] and followed a pre-planned, but unpublished protocol enclosed in the Supplementary Material.

### 2.1. Data Sources and Searches

Two investigators (NV and DP) independently conducted a literature search using several databases including PubMed/Medline, EMBASE, EBSCO, Web of Science from database inception until 23 November 2021, including RCTs investigating the effect of oral Mg vs. placebo on serum inflammatory parameters (outcome).

In PubMed, the following search strategy was used: (‘magnesium’) AND (‘inflammation’ OR ‘inflammatory’ OR ‘interferons’ OR ‘interferon’ OR ‘TNF’ OR ‘tumor necrosis factor’ OR ‘IL’ OR ‘interleukin’ OR ‘TGF’ OR ‘transforming growth factor’ OR ‘CRP’ OR ‘C-reactive protein’ OR ‘cytokines’ OR ‘cytokine’) AND (‘clinical trial’ OR ‘randomized controlled trial’ OR ‘placebo’), adapting the search according to the database. Any inconsistencies were resolved by consensus with a third author (LS).

### 2.2. Study Selection

Inclusion criteria for this meta-analysis were: (i) RCT; (ii) double-blind design; (iii) use of oral Mg supplementation; (iv) assessment of serum inflammatory parameters at follow-up evaluation; (v) written in English. Studies were excluded if: (i) did not include humans; (ii) used a control group taking other substances than placebo; (iii) lack of sufficient information regarding serum inflammatory parameters.

### 2.3. Data Extraction

Two independent investigators (NV and DP) extracted key data from the included articles in a standardized Excel spread sheet and a third independent investigator (LS) checked the data. For each article, we extracted data on author names, year of publication, country, condition, study design (crossover or parallel), Mg daily dosage, and follow-up duration (in weeks). Moreover, we extracted data by Mg or placebo in relation to mean age, body mass index (BMI), and number of females at baseline.

### 2.4. Outcomes

The primary outcomes were the values of serum parameters of inflammatory markers after treatment with Mg compared to placebo.

### 2.5. Quality Assessment

Two authors (NV and DP) completed scoring using the risk of bias (RoB) tool suggested by the Cochrane group [19]. This tool assesses several domains of the quality of each RCT, including: adequacy of random sequence generation, allocation concealment, blinding of participants, personnel and outcome assessors, incomplete data outcome (assessment of dropouts), selective outcome reporting, and the presence of other sources of bias. The potential answers were, as the Cochrane Handbook suggests, low risk of bias, high or unclear [20].

### 2.6. Data Synthesis and Analysis

All analyses were performed using STATA version 14.0 (StataCorp, College Station, TX, USA). Outcomes with at least three studies were meta-analyzed, whilst outcomes with less than three studies were reported descriptively.

The primary analysis compared serum parameters of inflammatory markers between participants treated with oral Mg supplementation vs. placebo at the follow-up evaluation. We calculated the difference between the means of the treatment and placebo groups using follow-up data through standardized mean differences (SMD) with their 95% confidence intervals (CIs), applying a random-effect model [21]. Heterogeneity across studies was assessed by the I^2^ metric and χ^2^ statistics. Given significant heterogeneity (I^2^ ≥ 50%, *p* < 0.05) and for outcomes having at least ten studies, we conducted a series of meta-regression analyses, according to follow-up (weeks), daily Mg dose, and differences at the baseline evaluation between treated with Mg and placebo in mean BMI, age, CRP serum levels, and percentage of females.

Publication bias was assessed by visually inspecting funnel plots and using the Begg–Mazumdar Kendall tau [22] and the Egger bias test [23].

For all analyses, a *p*-value less than 0.05 was considered statistically significant.

## 3. Results

### 3.1. Search Results

**Figure 1:**
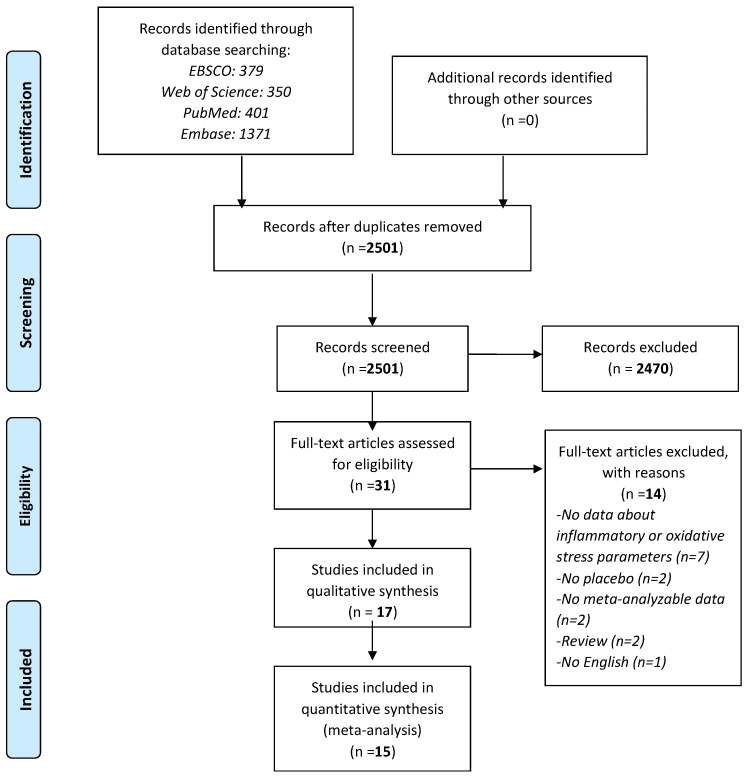
PRISMA flow-chart.

As shown in Figure 1, among 2501 records initially screened, 31 were retrieved as full-texts: of them, 17 papers [17,24,25,26,27,28,29,30,31,32,33,34,35,36,37,38,39] were included in the systematic review with 15 contributing to the meta-analysis.

### 3.2. Study and Patient Characteristics

Full details regarding descriptive findings are reported in Table 1.

Among the 17 RCTs included, six were conducted in Asia, eight in North or South America, and three in Europe. The conditions ranged from metabolic disorders (including diabetes, pre-diabetes, overweight/obesity) present in 12 RCTs, pregnancy (*n* = 1), cardiovascular (*n* = 1), and respiratory conditions (*n* = 2). The median follow-up was 12 weeks, with a range between 4 and 26. The majority of the studies (*n* = 7) used a quantity of 250 mg/daily of Mg oxide (*n* = 5).

Altogether, 447 participants were randomized to Mg treatment: these participants had a mean age of 47.1 ± 9.3 years, were mainly female (=62.5%) with a mean BMI of 29.0 kg/m^2^. Conversely, 442 participants were randomized to the placebo group, having a similar mean age (46.8 ± 8.7 years), % of females (59.6%), and mean BMI (29.2 kg/m^2^) to the intervention group (Table 1).

### 3.3. Meta-Analysis of Mg Supplementation versus Placebo on Serum Inflammatory Parameters

**Figure 2:**
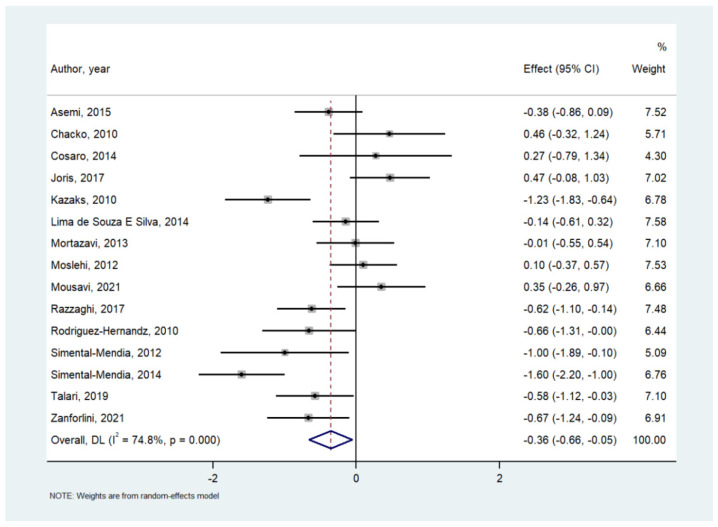
Forrest plot of the effect of magnesium versus placebo on serum C-reactive protein [17,24,25,26,27,28,29,30,31,32,33,35,36,38,39].

**Figure 3:**
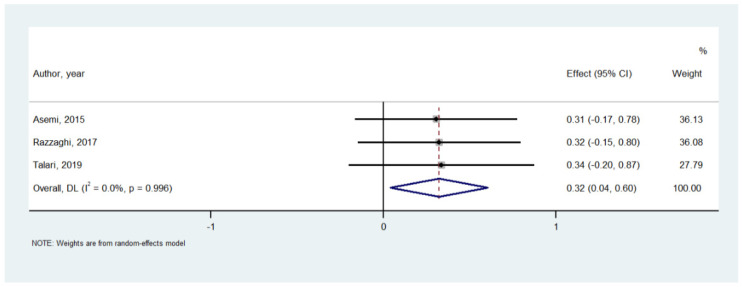
Forrest plot of the effect of magnesium versus placebo on serum nitric oxide [17,32,33].

Table 2 shows the effect of Mg on serum inflammatory parameters. Among 737 participants in 15 RCTs, compared to placebo, Mg supplementation significantly decreased serum CRP (SMD = −0.356; 95% CI: −0.659 to −0.054; *p* = 0.02) (Figure 2), although a high heterogeneity (I^2^ = 74.8%) was observed. Similarly, Mg supplementation increased nitric oxide levels (*n* = 3 studies; 194 participants; SMD = 0.321; 95% CI: 0.037 to 0.604; *p* = 0.026; I^2^ = 0%) (Figure 3). On the contrary, in the meta-analyses performed with at least three studies, Mg supplementation did not affect the serum levels of IL-6, total antioxidant capacity, glutathione (GSH), tumor necrosis factor alpha, whilst the effect on malondialdehyde (MDA) was at the limits of statistical significance (SMD = −0.604; 95% CI: −1.224 to 0.017; *p* = 0.057; I^2^ = 77.8%) (Table 2). Visual inspection of funnel plots and the Begg–Mazumdar Kendall tau and the Egger bias tests did not suggest the presence of publication bias.

Among the inflammatory parameters having less than three RCTs, Mg supplementation significantly reduced plasma fibrinogen, tartrate-resistant acid phosphatase type 5, tumor necrosis factor ligand superfamily member 13B, Tumorigenicity 2 protein, and IL(interleukin)-1, while no significant variations were observed on IL-8, IL-10, IL-17, erythrocyte sedimentation rate, and serum amyloid.

### 3.4. Risk of Bias

The risk of bias assessment is fully reported in Supplementary Table S1. In general, the risk of bias was generally low. Only one RCT [35] had a suspicious high risk of bias in sequence generation, allocation concealment and blinding of participants, personnel and outcome assessors. However, in the sequence generation 7/17, in the allocation concealment and in blinding of participants, personnel and outcome assessors 3 RCTs over 17 were at unclear risk of bias.

### 3.5. Meta-Regression Analysis

Supplementary Table S2 reports the data of the meta-regression taking the difference between treated with Mg and treated with placebo in serum CRP at the follow-up evaluation, as outcome. The only factor that was able to explain the heterogeneity of this outcome (I^2^ = 75.6%) was the difference in the percentage of women between the two groups (beta = 0.06; 95% CI: 0.004 to 0.11; *p* = 0.03) meaning that each increase in one percentage point in the difference of women between groups corresponded to an increase in 0.06 units of CRP (R^2^ = 39%).

## 4. Discussion

The present meta-analysis including 17 RCTs with more than 800 participants found that, when compared to placebo, Mg supplementation significantly reduced serum CRP levels, thus supporting previous literature [14,15]. In addition, we found that Mg supplementation increased NO levels.

Considering the inflammatory markers, assessed at least from three RCTs, no other significant variations were reported comparing the treated vs. placebo groups. Interestingly, although not statistically significant, three such important markers IL-6, GSH and MDA showed a reduction in treated groups compared to the baseline indicating that further studies are required to confirm these findings and render possible the formulation of stronger conclusions. In particular, it is important to understand the effects of Mg intake on IL-6 levels [40], which is secreted by T cells and macrophages and acts as both a pro-inflammatory and an anti-inflammatory cytokine. Therefore, IL-6 may be both an indicator of acute inflammation, or undetected infection [40]. In addition, as this topic is attracting increasing attention, recent evidence reported that Mg supplementation significantly improved the reduction of plasma fibrinogen [36,41], tartrate-resistant acid phosphatase type 5, TNF ligand superfamily member 13B, ST2 protein, and IL-1 [34,42].

Moreover, it has been reported that Mg deficiency, in animal models, may increase the recruitment of phagocytic cells to perform their effector functions, which ultimately leads to the generation of reactive oxygen species leading to an increased production of several cytokines involved in the inflammatory cascade, such as TNF-α [43]. At the same time, the release of these cytokines is induced by an increased level of intracellular Ca^2+^, which is considered a signal to start the inflammatory process and this condition could occur in case of Mg deficiency [44]. Moreover, other in animal and in vitro studies have indicated that pro-inflammatory cytokine production induced in case of Mg deficiency involves the pathway of NFκβ, with a consequent higher production of TNF-α and IL-1β [45]. Therefore, it is likely that all the systems involved in Mg deficiency may affect the inflammatory response in several ways and, in particular, through a modulation of intracellular calcium that regulates several pathways involved in inflammation [42].

Furthermore, this is the first meta-analysis showing that Mg can improve NO levels. This find could be crucial not only in terms of inflammatory mechanisms, but also from a cardiovascular point of view with a potential clinical impact. Interestingly, there is evidence showing that low Mg levels are associated with increased atrial fibrillation and coronary heart disease risk, while Mg supplementation is implied in the secondary prevention of cardiac arrhythmias [46,47]. Indeed, in vitro and in vivo animal studies have discovered a number of new electrophysiological properties of NO [48].

Finally, another interesting result of our systematic review and meta-analysis is that in meta-regression analyses the difference in percentage of females at baseline between treated and placebo groups was associated with a higher effect of Mg supplementation on serum CRP levels. Whilst it is known that males and females have significantly different levels of Mg not only in serum, but also in other biological fluids [49], the exact reason of the effect of Mg being more efficacious in females than in males in lowering serum CRP levels is not known and, therefore, future research is warranted.

The results of this meta-analysis should be considered taking in account its limitations. First, the RCTs included were small in sample size, had a limited follow-up time, and used different doses and types of Mg; therefore, the clinical applicability of our findings should be confirmed in higher quality RCTs. Second, several studies did not include the assessment of Mg introduced in the diet. However, it may be hypothesized that no significant difference in this parameter was present between treated and placebo groups due to randomization. The role of dietary Mg on the effect of Mg supplementation should be better determined. Third, for several outcomes, we were not able to run a meta-analysis since these outcomes were included in less than 3 studies. Finally, when considering the outcome with the largest number of studies, i.e., serum CRP, it was characterized by a high heterogeneity that we were able to explain only partly with the meta-regression analyses.

## 5. Conclusions

This systematic review and meta-analysis showed the beneficial effects of Mg supplementation in significantly reducing different inflammatory markers, in particular CRP and increasing NO levels. These data open new scenarios in clinical practice suggesting the importance of considering Mg supplementation in patient categories, with particular focus on cardiovascular diseases. Considering the presence of inconsistent but potentially useful data regarding Mg supplementation, further studies are desirable to corroborate and better clarify these findings.

## Figures and Tables

**Table 1:** Descriptive findings of the randomized controlled trials included.

							Magnesium				Placebo			
Author, Year	Country	Condition	Inflammatory Parameters	Daily Mg Doses (mg)	Type of Mg	Follow-Up (Weeks)	Sample Size	Age (SD) (Years)	Women (%)	BMI (SD)	Sample Size	Age (SD) (Years)	Women (%)	BMI (SD)
Alonso, 2020 [34]	USA	Cardiovascular Diseases	CRP, NO, TAC, GSH, MDA, Tartrate-resistant acid phosphatase type 5, ST2 protein, Interleukin-1 receptor type 1	400	Oxide	10	24	62 ± 5	88	28.3 ± 5.01	28	62 ± 6	61	27.8 ± 4.2
Asemi, 2015 [17]	Iran	Pregnancy	CRP, NO, TAC, MDA	250	Oxide	6	35	29.1 ± 4.6	100	29.6 ± 5.4	35	29.4 ± 3.1	100	29.1 ± 3.5
Chacko, 2010 [25]	USA	Overweight	CRP, IL-6, TNF-alfa	500	Citrate	4	13	47 ± 13.8	43	28.3 ± 1.6	13	41.9 ± 12.7	24	28.1 ± 2.2
Cosaro, 2014 [28]	Italy	Family history of metabolic syndrome	CRP	368	Pidolate	8	8				6			
Hosseini, 2016 [37]	Iran	Asthma	IL-17	340	Citrate	8	50	36.38 ± 9.72	50	25.6 ± 3.8	50	34.56 ± 8.28	44	26.19 ± 3.69
Joris, 2017 [31]	The Netherlands	Overweight/ obese	CRP, IL-6, IL-8, TNF-alfa, amyloid	350	Citrate	24	26				25			
Kazaks, 2010 [24]	USA	Asthma	CRP	340	Citrate	26	27	37 ± 2	50	29 ± 1	25	37 ± 2	61.1	28 ± 1
Lima de Souza E Silva, 2014 [29]	Brasil	Metabolic Syndrome	CRP	400	Chelate	12	35	44.6 ± 9.7		35.5 ± 8.2	37	46.6 ± 12.3		35.1 ± 6.3
Mortazavi, 2013 [27]	USA	Hemodialysis patients	CRP	440	Oxide	24	27	56.93 ± 12.19	48.3		25	56.36 ± 11.15	48	
Moslehi, 2012 [36]	Iran	Overweight	CRP, IL-6, fibrinogen	250	Oxide	8	35		100	27.9 ± 3.2	34		100	27.9 ± 3
Mousavi, 2021 [39]	Iran	Polycystic ovary syndrome	CRP, TAC, MDA, TNF-alfa	250	Oxide	8	21	25.6 ± 4.9	100	28.0 ± 3.2	20	26.2 ± 5.7	100	26.9 ± 3.8
Razzaghi, 2018 [32]	Iran	Diabetic foot ulcer	CRP, NO, TAC, GSH, MDA, ERS	250	Oxide	12	35	60.1 ± 11.1	37.1	28.2 ± 5.2	35	59 ± 10.1	31.4	26.3 ± 4.2
Rodriguez-Hernandez, 2010 [35]	Mexico	Obese	CRP	450	Chloride	16	19	44.2 ± 10.8	63.6	30.5 ± 4.4	19	43.2 ± 7.8	63.6	35.1 ± 7.9
Simental-Mendia, 2012 [26]	Mexico	Prediabetes	CRP, IL-6, IL-10, TNF-alfa	382	Chloride	12	11	44.2 ± 10.8	63.6	30.5 ± 4.4	11	43.2 ± 7.8	63.6	35.1 ± 7.9
Simental-Mendia, 2014 [30]	Mexico	Prediabetes	CRP	382	Chloride	12	29	39.8 ± 16	55.2	30.5 ± 5.7	28	41.1 ± 13.1	60.7	30 ± 5.7
Talari, 2019 [33]	Iran	Diabetic hemodialysis	CRP, NO, TAC, GSH, MDA	250	Oxide	24	27	58.8 ± 10.1	51.9	27.2 ± 5.6	27	61.8 ± 10.2	55.6	26.2 ± 4.4
Zanforlini, 2021 [38]	Italy	Chronic obstructive pulmonary disease	CRP, TNF-alfa	300	Citrate	24	21	73 ± 8.9	24	26.9 ± 4.3	20	72.2 ± 11	20.8	26.9 ± 3.8
Total						Median = 12	447	47.1 ± 9.3	62.5	29.0 ± 4.4	442	46.8 ± 8.7	59.6	29.2 ± 4.4

**Table 2:** Meta-analysis of magnesium supplementation on serum inflammatory parameters.

Inflammatory Parameter	Number of Comparisons	Number of Participants	SMD	95% CI		*p* Value	I^2^	Egger’s Test (*p*-Value)
CRP	15	737	−0.356	−0.659	−0.054	0.02	74.8	−0.28 (0.92)
IL-6	3	142	−0.258	−1.083	0.567	0.54	81.3	0.94 (0.38)
NO	3	194	0.321	0.037	0.604	0.03	0	0.67 (0.40)
TAC	4	235	0.189	−0.491	0.869	0.59	84.8	8.86 (0.53)
GSH	3	194	−0.181	−0.463	0.102	0.21	0	3.00 (0.61)
MDA	3	194	−0.604	−1.224	0.02	0.06	77.8	−13.9 (0.68)
TNF-a	3	112	0.168	−0.433	0.768	0.58	58.8	3.84 (0.68)

## Data Availability

The data and the databases are available upon reasonable request to the Corresponding Author.

## Supplementary Materials

The following supporting information can be downloaded at: https://www.mdpi.com/article/10.3390/nu14030679/s1, supplementary Table S1: Risk of bias in the randomized controlled trials included; supplementary Table S2: Meta-regression analyses.
