# Supplementary material for: Effect of Magnesium Supplementation on Inflammatory Parameters: A Meta-Analysis of Randomized Controlled Trials
Source: Nutrients. 2022 Feb 5;14(3):679. doi: 10.3390/nu14030679 (PMC8838086; doi:10.3390/nu14030679)
Supplement: Supplementary file 1 [file nutrients-14-00679-s001.zip › nutrients-1550861-supplementary.pdf]

Supplementary material. Protocol

Effect of Magnesium Supplementation on Inflammatory Parameters: a Meta-Analysis of Randomized Controlled Trials

Author: Dr. Nicola Veronese

Brief rationale

The literature regarding the health benefits of magnesium (Mg) is exponentially increasing.[1] An umbrella review with 16 meta-analyses and 50 independent outcomes findings suggested that Mg is associated with several positive health outcomes.[1] It is widely known that Mg is involved in more than 600 enzymatic reactions[2], consequently having a wide spectrum of actions in pregnancy[3-5], as well as in cardiovascular[6,7], gastrointestinal[8], infectious,[9] and metabolic diseases[10], such as diabetes. [11,12]

All these health conditions have in common low-grade inflammation. To know if Mg could reduce this condition could be useful for clinical purposes.

The present systematic review and meta-analysis will aim to summarize the current state of the art of all randomized control trials (RCTs) investigating the effects of Mg supplementation versus placebo on serum parameters of chronic inflammation.

Review Protocol

Search

We will independently conducte a literature search using several databases including PubMed, EMBASE, EBSCO, Web of Science from database inception, including RCTs investigating the effect of oral Mg vs placebo on serum inflammatory parameters (outcome).

In PubMed, the following search strategy will be used: (‘magnesium’) AND (‘inflammation’ OR ‘inflammatory’ OR ‘interferons’ OR ‘interferon’ OR ‘TNF’ OR ‘tumor necrosis factor’ OR ‘IL’ OR ‘interleukin’ OR ‘TGF’ OR ‘transforming growth factor’ OR ‘CRP’ OR ‘C-reactive protein’ OR ‘cytokines’ OR ‘cytokine’) AND (‘clinical trial’ OR ‘randomized controlled trial’ OR ‘placebo’), adapting the search according to the database. Any inconsistencies were resolved by consensus with a third senior author.

We will set the databases so the search covers titles, abstracts, and key words.

Inclusion/ Exclusion Criteria

Inclusion criteria for this meta-analysis will be: (i) RCT; (ii) double-blind design; (iii) use of oral Mg supplementation; (iv) assessment of serum inflammatory parameters at follow-up evaluation; (v) written in English. Studies will be excluded if: (i) did not include humans; (ii) used a control group taking other substances than placebo; (iii) lack of sufficient information regarding serum inflammatory parameters.

Screening

First titles of all articles returned from the search will be screened by one reviewer. Following title screen, abstracts of remaining papers will be screened by two independent reviewers, and any disagreement will be resolved by a third senior reviewer. Following abstract screen, full-text of remaining papers will be screened by two independent reviewers, and any disagreement will be resolved by a third reviewer. At each stage screening will take place in line with the inclusion and exclusion criteria.

After screening is completed all reference lists of included papers will be searched to identify any missing papers and so on.

Data Extraction

The following data will be extracted from each paper: authors, year of publication, country, condition, study design (crossover or parallel), Mg daily dosage, and follow-up duration (in weeks). Moreover, we extracted data by Mg or placebo in relation to mean age, body mass index (BMI), and number of females at baseline.

Quality Assessment

Two independent authors will complete scoring using the risk of bias (RoB) tool suggested by the Cochrane group.**[13]** This tool assesses several domains of the quality of each RCT, including: adequacy of random sequence generation, allocation concealment, blinding of participants, personnel and outcome assessors, incomplete data outcome (assessment of dropouts), selective outcome reporting, and the presence of other sources of bias. The potential answers were, as the Cochrane Handbook suggests, low risk of bias, high or unclear. **[14]**

Statistical Analyses

All analyses will be performed using STATA version 14.0 (StataCorp) by one author, expert of the field. Outcomes with at least three studies will be meta-analyzed, whilst outcomes with less than three studies will be reported descriptively.

The primary analysis will compare serum parameters of inflammatory markers between participants treated with oral Mg supplementation vs. placebo at the follow-up evaluation. We will calculate the difference between the means of the treatment and placebo groups using follow-up data through standardized mean differences (SMD) with their 95% confidence intervals (CIs), applying a random-effect model.**[15]** Heterogeneity across studies will ve assessed by the I^2 metric and χ^2 statistics. Given significant heterogeneity ($I^2 \geq 50\%$, $P<0.05$) and for outcomes having at least ten studies, we will conduct a series of meta-regression analyses, according to follow-up (weeks), daily Mg dose, and differences at the baseline evaluation between treated with Mg and placebo in mean BMI, age, CRP serum levels, and percentage of females.

Publication bias will be assessed by visually inspecting funnel plots and using the Begg–Mazumdar Kendall tau**[16]** and the Egger bias test.**[17]**

For all analyses, a P-value less than 0.05 will be considered statistically significant, two-tailed.

Ethics and Final Report

This research is exempt from ethics approval because the work is carried out on published documents. The final report will be prepared following the Preferred Reporting Items for Systematic Reviews and Meta-Analyses (PRISMA) guidelines. It will be written up as a peer-reviewed publication and it will be submitted to a peer-reviewed academic journal such as the European Heart Journal or the British Medical Journal at the discretion of Ferring Pharmaceuticals.

Keywords: Magnesium; Inflammation

References

1. Veronese, N.; Demurtas, J.; Pesolillo, G.; Celotto, S.; Barnini, T.; Calusi, G.; Caruso, M.G.; Notarnicola, M.; Reddavide, R.; Stubbs, B. Magnesium and health outcomes: an umbrella review of

systematic reviews and meta-analyses of observational and intervention studies. *European Journal of Nutrition* **2020**, *59*, 263-272.

2. Caspi, R.; Altman, T.; Billington, R.; Dreher, K.; Foerster, H.; Fulcher, C.A.; Holland, T.A.; Keseler, I.M.; Kothari, A.; Kubo, A. The MetaCyc database of metabolic pathways and enzymes and the BioCyc collection of Pathway/Genome Databases. *Nucleic acids research* **2014**, *42*, D459-D471.
3. Kovacs, L.; Molnar, B.; Huhn, E.; Bodis, L. Magnesium substitution in pregnancy. A prospective, randomized double-blind study. *Geburtshilfe und Frauenheilkunde* **1988**, *48*, 595-600.
4. Bartal, M.F.; Sibai, B.M. Eclampsia in the 21st century. *American journal of obstetrics and gynecology* **2020**.
5. Fanni, D.; Gerosa, C.; Nurchi, V.; Manchia, M.; Saba, L.; Coghe, F.; Crisponi, G.; Gibo, Y.; Van Eyken, P.; Fanos, V. The role of magnesium in pregnancy and in fetal programming of adult diseases. *Biological Trace Element Research* **2021**, *199*, 3647-3657.
6. Fang, X.; Wang, K.; Han, D.; He, X.; Wei, J.; Zhao, L.; Imam, M.U.; Ping, Z.; Li, Y.; Xu, Y. Dietary magnesium intake and the risk of cardiovascular disease, type 2 diabetes, and all-cause mortality: a dose–response meta-analysis of prospective cohort studies. *BMC medicine* **2016**, *14*, 1-13.
7. Dominguez, L.; Veronese, N.; Barbagallo, M. Magnesium and Hypertension in Old Age. *Nutrients* **2021**, *13*, 139.
8. Gröber, U.; Schmidt, J.; Kisters, K. Magnesium in prevention and therapy. *Nutrients* **2015**, *7*, 8199-8226.
9. Dominguez, L.J.; Veronese, N.; Guerrero-Romero, F.; Barbagallo, M. Magnesium in Infectious Diseases in Older People. *Nutrients* **2021**, *13*, 180.
10. Barbagallo, M.; Veronese, N.; Dominguez, L.J. Magnesium in aging, health and diseases. *Nutrients* **2021**, *13*, 463.
11. Veronese, N.; Watutantrige-Fernando, S.; Luchini, C.; Solmi, M.; Sartore, G.; Sergi, G.; Manzato, E.; Barbagallo, M.; Maggi, S.; Stubbs, B. Effect of magnesium supplementation on glucose metabolism in people with or at risk of diabetes: a systematic review and meta-analysis of double-blind randomized controlled trials. *European journal of clinical nutrition* **2016**, *70*, 1354-1359.
12. Veronese, N.; Dominguez, L.J.; Pizzol, D.; Demurtas, J.; Smith, L.; Barbagallo, M. Oral Magnesium Supplementation for Treating Glucose Metabolism Parameters in People with or at Risk of Diabetes: A Systematic Review and Meta-Analysis of Double-Blind Randomized Controlled Trials. *Nutrients* **2021**, *13*, 4074.
13. Higgins, J.P.; Altman, D.G.; Gøtzsche, P.C.; Jüni, P.; Moher, D.; Oxman, A.D.; Savović, J.; Schulz, K.F.; Weeks, L.; Sterne, J.A. The Cochrane Collaboration's tool for assessing risk of bias in randomised trials. *Bmj* **2011**, *343*.
14. Higgins, J.P.; Thomas, J.; Chandler, J.; Cumpston, M.; Li, T.; Page, M.J.; Welch, V.A. *Cochrane handbook for systematic reviews of interventions*; John Wiley & Sons: 2019.
15. DerSimonian, R.; Laird, N. Meta-analysis in clinical trials. *Controlled clinical trials* **1986**, *7*, 177-188.
16. Begg, C.B.; Mazumdar, M. Operating characteristics of a rank correlation test for publication bias. *Biometrics* **1994**, 1088-1101.
17. Egger, M.; Smith, G.D.; Schneider, M.; Minder, C. Bias in meta-analysis detected by a simple, graphical test. *Bmj* **1997**, *315*, 629-634.

Supplementary Table S1. Risk of bias in the randomized controlled trials included.

Study	Sequence generation	Allocation Concealment	Blinding of participants, personnel and outcome assessors	Incomplete data outcome	Selective outcome reporting	Other sources of bias
Alonso, 2020	U	L	L	L	L	L
Asemi, 2015	U	L	L	L	L	L
Chacko, 2010	L	L	L	L	L	L
Cosaro, 2014	L	L	L	L	L	L
Hosseini, 2016	L	L	L	L	L	L
Joris, 2017	U	L	L	L	L	L
Kazaks, 2010	L	L	L	L	L	U
Lima de Souza E Silva, 2014	U	U	U	L	L	U
Mortazavi, 2013	L	L	L	L	L	L
Moslehi, 2012	L	L	L	L	L	L
Mousavi, 2021	L	L	L	U	L	L
Razzaghi, 2017	U	L	L	L	L	L
Rodriguez-Hernandez, 2010	H	H	H	L	L	U
Simental-Mendia, 2012	U	U	U	L	L	U
Simental-Mendia, 2014	U	U	U	L	L	U
Talari, 2019	L	L	L	L	L	L
Zanforlini, 2021	L	L	L	H	L	H

Abbreviations: U: unclear; L: low; H: high

Supplementary Table S2. Meta-regression analyses.

Parameter	Number of studies	beta	95% CI	R^2
**Follow-up (weeks)**	15	-0.02	-0.07-0.03	0.00
**Daily Mg dose**	15	0.0003	-0.004-0.005	0.00
**Difference in BMI (treated vs. placebo)**	12	0.05	-0.16-0.26	0.00
**Difference in CRP at baseline (treated vs. placebo)**	15	0.04	-0.84-0.38	0.00
**Difference in mean age (treated vs. placebo)**	12	0.09	-0.11-0.30	0.00
**Difference in % of women (treated vs. placebo)**	**12**	**0.06**	**0.004-0.11**	**39.3**

Abbreviations: body mass index: BMI; C reactive protein: CRP.
